# PD69 Road Map Of Health Equity Considerations In Health Technology Assessments: Identifying The Evidence From Epidemiology To Health Economics

**DOI:** 10.1017/S026646232400326X

**Published:** 2025-01-07

**Authors:** Grammati Sarri, Amruta Radhakrishnan, Lydia Vinals

## Abstract

**Introduction:**

Achieving health equity through access to new health technologies has not been consistently considered within the health technology assessment (HTA) remit. HTA bodies now recognize that achieving health equity (or reducing health inequities) is a distinct value element for new technologies beyond the restrictive focus on clinical benefits and costs, but they lack familiarity with methods to reliably measure such effects.

**Methods:**

We conducted a targeted literature review in indexed databases (Embase and MEDLINE) to identify relevant literature on methods for measuring health equity in HTAs published in the English language within the last five years. We mapped the literature based on key HTA stages: topic selection (eligible patient population); scoping (description of pre-decision health equities); evidence synthesis (equity impacts on health outcomes from introducing the intervention); comparative clinical comparisons including indirect treatment comparisons and cost-effectiveness analyses (including assessment of equity and efficiency trade-offs); and re-assessment (for conditional marketing). Equity-informed HTA decision frameworks and checklists were separately summarized. A narrative synthesis was performed.

**Results:**

The databases returned 2,800 unique publications. After screening 50 percent of the abstracts (30 publications) were found to be relevant. We applied additional selection criteria based on quality and information availability. Findings across the studies were grouped in a health equity methodological map as follows: understanding baseline epidemiological data in the targeted population; assessing diversity in trial populations and need for additional robust real-world evidence; effectiveness methods (subgroup analyses, incorporation of observational data with trials, simulation studies); and expanded economic modeling (distributional cost-effectiveness analysis, augmented cost-effectiveness analysis, extended cost-effectiveness analysis) or the use of multicriteria decision analysis. Two equity checklists and frameworks for HTA were also identified.

**Conclusions:**

Greater familiarity with health equity methods will drive its routine implementation in HTA and decision-making. This road map provides a summary of available health equity methodologies across the full HTA cycle and can be used as a guide for future HTA case studies. Decision-makers should directly engage with patients to learn about the experiences of diverse and disadvantaged patient groups.

